# Real-world pharmacogenetics of statin intolerance: effects of *SLCO1B1, ABCG2*, and *CYP2C9* variants

**DOI:** 10.1097/FPC.0000000000000504

**Published:** 2023-07-23

**Authors:** K. Ivar Lönnberg, Aleksi Tornio, Päivi Hirvensalo, Jenni Keskitalo, Anna-Liina Mustaniemi, Johanna I. Kiiski, Anne M. Filppula, Mikko Niemi

**Affiliations:** a Department of Clinical Pharmacology, University of Helsinki; b Individualized Drug Therapy Research Program, Faculty of Medicine, University of Helsinki, Helsinki; c Integrative Physiology and Pharmacology, Institute of Biomedicine, University of Turku; d Unit of Clinical Pharmacology, Turku University Hospital, Turku; e Department of Clinical Pharmacology, HUS Diagnostic Center, Helsinki University Hospital, Helsinki; f Pharmaceutical Science Laboratory Åbo Akademi University, Turku, Finland

**Keywords:** ABCG2, CYP2C9, intolerance, SLCO1B1, statin

## Abstract

The association of *SLCO1B1* c.521T>C with simvastatin-induced muscle toxicity is well characterized. However, different statins are subject to metabolism and transport also by other proteins exhibiting clinically meaningful genetic variation. Our aim was to investigate associations of *SLCO1B1* c.521T>C with intolerance to atorvastatin, fluvastatin, pravastatin, rosuvastatin, or simvastatin, those of *ABCG2* c.421C>A with intolerance to atorvastatin, fluvastatin, or rosuvastatin, and that of CYP2C9*2 and *3 alleles with intolerance to fluvastatin.

**Methods:**

We studied the associations of these variants with statin intolerance in 2042 patients initiating statin therapy by combining genetic data from samples from the Helsinki Biobank to clinical chemistry and statin purchase data.

**Results:**

We confirmed the association of *SLCO1B1* c.521C/C genotype with simvastatin intolerance both by using phenotype of switching initial statin to another as a marker of statin intolerance [hazard ratio (HR) 1.88, 95% confidence interval (CI) 1.08–3.25, *P* = 0.025] and statin switching along with creatine kinase measurement (HR 5.44, 95% CI 1.49–19.9, *P* = 0.011). No significant association was observed with atorvastatin and rosuvastatin. The sample sizes for fluvastatin and pravastatin were relatively small, but *SLCO1B1* c.521T>C carriers had an increased risk of pravastatin intolerance defined by statin switching when compared to homozygous reference T/T genotype (HR 2.11, 95% CI 1.01–4.39, *P* = 0.047).

**Conclusion:**

The current results can inform pharmacogenetic statin prescribing guidelines and show feasibility for the methodology to be used in larger future studies.

## Introduction

Lipid-lowering 3-hydroxy-3-methylglutaryl-coenzyme-A reductase inhibitors (statins) are among the most widely used drugs in the world. They are used both in primary and secondary prevention to reduce cardiovascular risk. Even though statins are generally well tolerated, their most typical adverse effects include muscular toxicity ranging from relatively common muscle pain (myalgia) to very rare but potentially life-threatening rhabdomyolysis [1]. Statin-induced muscle toxicity is a dose- and concentration-dependent phenomenon [2,3], and several predisposing factors due to increased statin concentrations have been identified, including drug-drug interactions and genetic factors [4–7].

Statins are similar in their pharmacodynamic effects, but their pharmacokinetic properties exhibit significant differences [8,9]. Simvastatin and lovastatin are administered as inactive lactones and are converted to an active acid form, whereas other statins in clinical use (atorvastatin, fluvastatin, pitavastatin, pravastatin, and rosuvastatin) are administered in the active acid form. The main clearance mechanism for the more lipophilic statins is oxidative biotransformation by cytochrome P450 (CYP) 3A4 (atorvastatin, lovastatin, and simvastatin) and CYP2C9 (fluvastatin), whereas the more hydrophilic statins (pitavastatin, pravastatin and rosuvastatin) are excreted mainly unchanged. Furthermore, all statins are transported by organic anion transporting polypeptide (OATP) 1B1 (encoded by *SLCO1B1*) and atorvastatin, fluvastatin and rosuvastatin by breast cancer resistance protein (BCRP, encoded by *ABCG2*) [5,8,10]. Accordingly, genetic variability affecting the function of drug-metabolizing enzymes and transporters can have a profound impact on statin pharmacokinetics, translating into altered risk for concentration-dependent adverse effects between individuals at equal doses.

A recently published Clinical Pharmacogenetics Implementation Consortium guideline on the pharmacogenetics of statin-induced muscle symptoms provided therapeutic recommendations for statins based on *SLCO1B1, ABCG2*, and *CYP2C9* genotypes [11]. Simvastatin acid is among the most sensitive drugs to alterations in *SLCO1B1* genotype [5,6,12–14]. Moreover, *SLCO1B1* c.521T>C has been consistently associated with simvastatin and atorvastatin-induced myopathy and intolerance, especially when high doses are used [5,7,12–17]. Although the pharmacokinetic effects are clear, the clinical evidence linking genetic variants in other pharmacokinetic genes as well as statins other than simvastatin and atorvastatin with statin intolerance is sparse. Thus, we sought to study the effects of *SLCO1B1, ABCG2* and *CYP2C9* genotypes on statin intolerance in a large cohort of incident statin users linked to biobank and national register data.

## Methods

### Study design and data sources

This was a register-based cohort study linking data from the Helsinki Biobank with Finnish national health registries to investigate pharmacogenomics of statins. Helsinki Biobank identified eligible individuals (see Study population). Information on drug purchases was collected from the drug reimbursement registry maintained by the Social Insurance Institution of Finland (Kela). The registry contains the identification code for drug purchased (ATC code), the date of purchase, the purchased amount in milligrams, the size of the package, and number of packages purchased covering all residents in Finland. In addition to first statin purchase, all subsequent statin purchases were collected from the period 1 January 1998 to 31 December 2016. Information about plasma creatine kinase (CK) measurements and date of measurements, was provided by Helsinki University Hospital Laboratory Services (HUSLAB). Helsinki Biobank linked information from these sources with pseudonymized identifiers assigned to every individual in the study. The biobank also provided pseudonymized DNA samples from all subjects for genotyping.

### Study population

The study population consisted of patients who had initiated statin therapy between 1998 and 2016. Individuals fulfilling the following inclusion criteria were included in the study: (1) at least one purchase of atorvastatin, fluvastatin, rosuvastatin, pravastatin, or simvastatin during the period between 1 January 1998 and 31 December 2016, (2) no purchase of a statin during 1997 and (3) a signed biobank consent and a DNA sample available in the Helsinki Biobank for analysis. Pitavastatin and lovastatin were not included because pitavastatin is not available in Finland and lovastatin usage is minimal. The first statin purchased by the study patient was defined as the index statin. Individuals were excluded from analysis based on the following criteria: (1) index statin was a simvastatin and ezetimibe combination product, (2) index statin was atorvastatin or rosuvastatin which was switched to another statin between May 2006 and April 2007 or (3) missing relevant genotype data. The drug reimbursement policy in Finland changed in 2006 so that atorvastatin and rosuvastatin were reimbursed only if other statins were not tolerated or not sufficiently effective. This led to excessive number of switches unrelated to statin intolerance from atorvastatin or rosuvastatin to other statins between May 2006 and April 2007. One subject withdrew the biobank consent during the study and was excluded from the analyses. Follow-up started from the first purchase of the index statin and the study patients were censored from the analyses when they had no subsequent statin purchases during 365 days, they died, or the follow-up time ended (31 December 2016). Daily statin dose was assumed to be one unit per day [18].

### Statin intolerance phenotypes

Primary statin intolerance phenotype was defined as a switch to another statin from the initially purchased index statin. Intolerance event date was defined as the last purchase date of the index statin before the switch. ATC code was used as an identifier for the drug used and thus change to another brand was not considered as a statin switch. Secondary intolerance phenotype was switch as defined in the primary intolerance phenotype combined with temporal relation to CK value measurement. Temporal relation was defined as CK measurement occurring within a period of 30 days before to 90 days after the intolerance event date. The CK measurement was considered as a binary outcome and the numeric value of the CK measurement was not considered in the analyses. Each intolerance phenotype was only analyzed if there were more than 10 occurrences of the phenotype for an index statin. Stata 16.1 (StataCorp LLC, College Station, Texas, USA) was used for defining the statin intolerance phenotypes.

### Genotyping

DNA samples were provided by the Helsinki Biobank at the concentration of 50 ng/µl. The participants were genotyped for the *ABCG2* rs2231142 (c.421C>A, p.Gln141Lys), *SLCO1B1* rs4149056 (c.521T>C, p.Val174Ala), and the *CYP2C9* rs1799853 (c.430C>T, p.Arg144Cys, *2) and rs1057910 (c.1075A>G, p.Ile359Leu, *3) single nucleotide variations (SNV) using TaqMan genotyping assays on OpenArray plates and the QuantStudio 12K Flex Real-Time PCR System (Thermo Fisher Scientific, Waltham, Massachusetts, USA). Genotyping results were interpreted using TaqMan Genotyper Software v.1.3 (Thermo Fisher Scientific). Homozygotes or compound heterozygotes for CYP2C9*2 or *3 were classified as poor CYP2C9 metabolizers (PM), heterozygotes as intermediate metabolizers, and non-carriers as normal metabolizers.

### Statistical analysis

Cox proportional hazards regression models were used to investigate possible associations of genetic variants with the occurrence of the statin intolerance phenotypes. Daily statin doses were set as covariates in the analyses as categorical variables. Genotypes were analyzed as categorical variables using an additive model. In case the variant homozygous genotype group or the lowest or highest dose group contained less than 10 individuals, the group was joined with the adjacent group. The comparisons were carried out for individual SNVs, CYP2C9 metabolizer classes, or for the numbers of risk, that is, variant alleles in the candidate genes for a specific statin including the statin dose in the model. Hardy-Weinberg equilibrium *P*-values were approximated with 10 000 permutations. The statistical analyses were carried out with IBM SPSS Statistics 27 (IBM Corp., Armonk, New York, USA) and JMP Genomics 8.2 (SAS Institute Inc., Cary, North Carolina, USA).

### Ethical aspects

The study was conducted in accordance with the Declaration of Helsinki. The ethics committee of the Hospital District of Helsinki and Uusimaa approved the study (HUS/2926/2017). Permission to use the data and samples was granted by the Hospital District of Helsinki and Uusimaa (HUS/359/2017) and permissions to use the prescription data were obtained from the Social Insurance Institution of Finland (Kela 9/522/2018) and the clinical chemistry data from the Finnish Institute for Health and Welfare (THL/161/5.05.00/2018). The research permit was granted by the Hospital District of Helsinki and Uusimaa (HUS 32/2018). After record linkage in the Helsinki Biobank, the data was provided to the study group in a pseudonymized form. The patients had given informed consent to Helsinki Biobank for their samples and data to be used for research purposes as defined in the Finnish Biobank Act.

## Results

**Fig. 1:**
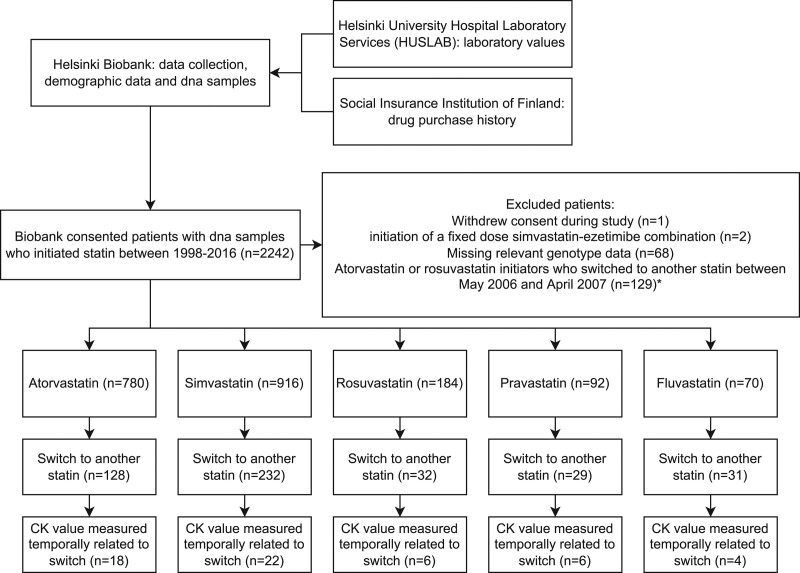
Data collection process, exclusions, and distribution of participants according to the statin initiated. *The drug reimbursement policy in Finland changed in 2006 so that atorvastatin and rosuvastatin were reimbursed only if other statins were not tolerated or not sufficiently effective.

Data and DNA samples were obtained from 2242 biobank consented patients who had initiated statin therapy between 1998 and 2016 (Fig. 1). A total of 200 patients were excluded from analysis due to one of the following reasons: withdrawal of biobank consent (n = 1), a fixed-dose simvastatin-ezetimibe combination as the index therapy (n = 2), unsuccessful genotyping of relevant variants (n = 68), or switch from atorvastatin or rosuvastatin to another statin in the time between May 2006 and April 2007 (n = 129). The final study sample consisted of 2042 participants of whom 1073 (52.5%) were women. Their mean ± SD age was 60 ± 11.8 years. Genotyping call rate ranged between 0.94 and 0.99 and the observed genotype frequencies were in the Hardy-Weinberg equilibrium (Table 1). The observed minor allele frequencies were nearly identical to those observed in the Finnish population previously but differed slightly from previous data in the European population [19,20].

**Table 1:** Investigated single nucleotide variants and their frequencies among the 2241 Finnish patients

Gene	rsID	Nucleotide change	Amino acid change	Heterozygotes	Homozygotes	HWE, *P*-value	Call rate	MAF present study	MAF Finnish^a^	MAF Europeans^b^
*ABCG2*	rs2231142	c.421C>A	p.Gln141Lys	0.13	0.007	0.26	0.99	0.07	0.07	0.08
*SLCO1B1*	rs4149056	c.521T>C	p.Val174Ala	0.32	0.043	0.65	0.97	0.20	0.21	0.16
*CYP2C9*	rs1799853	c.430C>T	p.Arg144Cys	0.19	0.016	0.08	0.94	0.11	0.11	0.12
*CYP2C9*	rs1057910	c.1075A>G	p.Ile359Leu	0.12	0.003	0.71	0.98	0.06	0.06	0.07

HWE, Hardy-Weinberg equilibrium; MAF, minor allele frequency.

a Finnish MAF is from the Sequencing Initiative Suomi project (SISu), Institute for Molecular Medicine Finland (FIMM), University of Helsinki, Finland (URL: http://sisuproject.fi) [SISu v4.1, accessed March 2023] [19].

b European MAF is from the 1000 Genomes project [20].

### Simvastatin

The median follow-up time for simvastatin initiators was 532 days. Of the total 916 simvastatin users, 232 were classified as statin switchers and of those, 22 had a temporally qualifying CK measurement (statin switchers with CK measurement; Fig. 1). In the Cox proportional hazards model accounting for the statin dose, *SLCO1B1* c.521C/C genotype was associated with an increased risk of simvastatin intolerance when compared to homozygous reference T/T genotype using both statin switcher [hazard ratio (HR) 1.88, 95% confidence interval (CI) 1.08–3.25, *P* = 0.025] and statin switcher with CK measurement (HR 5.44, 95% CI 1.49–19.9, *P* = 0.011) phenotypes (Table 2). Simvastatin dose of 40 mg or higher was also associated with the statin switcher phenotype when compared to 10 mg dose (HR 1.48, 95% 1.03–2.12, *P *= 0.035).

**Table 2:** Associations of *SLCO1B1* c.521T>C single nucleotide variation with intolerance to simvastatin

Variable	n	Statin switch			Statin switch + CK measurement		
		Events (n)	Hazard ratio (95% CI)	*P*-value	Events (n)	Hazard ratio (95% CI)	*P*-value
*SLCO1B1*							
10 mg dose	229	46			3		
20 mg dose	451	103	1.13 (0.80–1.60)	0.498	12	2.00 (0.56–7.14)	0.284
40 mg or 80 mg dose	236	83	1.48 (1.03–2.12)	0.035	7	1.84 (0.47–7.12)	0.379
c.521T/T	584	146			10		
c.521T/C	300	72	0.94 (0.70–1.24)	0.640	9	1.75 (0.71–4.31)	0.226
c.521C/C	32	14	1.88 (1.08–3.25)	0.025	3	5.44 (1.49–19.9)	0.011

CK, creatine kinase; CI, confidence interval.

### Atorvastatin

Atorvastatin had median follow-up time of 556.5 days. There were 780 atorvastatin users. Of those, 128 were categorized as statin switchers and 18 had a temporally qualifying CK measurement. Neither the *SLCO1B1* or *ABCG2* genotypes nor atorvastatin dose were associated with the statin intolerance phenotypes (Table 3).

**Table 3:** Associations of *SLCO1B1* c.521T>C and *ABCG2* c.421C>A single nucleotide variations with intolerance to atorvastatin

Variable	n	Statin switch			Statin switch + CK measurement		
		Events (n)	Hazard ratio (95% CI)	*P*-value	Events (n)	Hazard ratio (95% CI)	*P*-value
*SLCO1B1*							
10 mg dose	361	59			10		
20 mg dose	268	42	0.86 (0.58–1.28)	0.463	7	0.85 (0.32–2.24)	0.745
40 mg or 80 mg dose	151	27	1.03 (0.65–1.62)	0.907	1	0.22 (0.03–1.73)	0.151
c.521T/T	506	88			11		
c.521T/C	238	33	0.82 (0.55–1.23)	0.339	6	1.25 (0.46–3.38)	0.667
c.521C/C	36	7	1.13 (0.52–2.44)	0.759	1	1.30 (0.17–10.1)	0.799
*ABCG2*							
10 mg dose	361	59			10		
20 mg dose	268	42	0.85 (0.57–1.27)	0.428	7	0.86 (0.33–2.26)	0.759
40 mg or 80 mg dose	151	27	1.01 (0.64–1.60)	0.955	1	0.22 (0.03–1.75)	0.154
c.421C/C	675	110			15		
c.421C/A or A/A	105	18	1.04 (0.63–1.71)	0.877	3	1.27 (0.37–4.39)	0.706
Risk alleles							
10 mg dose	361	59			10		
20 mg dose	268	42	0.85 (0.57–1.27)	0.430	7	0.86 (0.33–2.27)	0.762
40 mg or 80 mg dose	151	27	1.02 (0.64–1.60)	0.949	1	0.23 (0.03–1.77)	0.157
0–1 alleles	705	117			17		
2–4 alleles	75	11	0.94 (0.51–1.74)	0.843	1	0.58 (0.08–4.39)	0.600

CK, creatine kinase; CI, confidence interval.

### Rosuvastatin

Rosuvastatin median follow-up time was 92.5 days. Rosuvastatin had total of 184 users of whom 32 were categorized as statin switchers. Rosuvastatin dose of 10 mg was associated with increased risk of statin intolerance when compared to 5 mg dose, but the *SLCO1B1* or *ABCG2* genotypes were not associated with statin intolerance (Table 4).

**Table 4:** Associations of *SLCO1B1* c.521T>C and *ABCG2* c.421C>A single nucleotide variations with intolerance to rosuvastatin

Variable	n	Statin switch		
		Events (n)	Hazard ratio (95% CI)	*P*-value
*SLCO1B1*				
5 mg dose	53	3		
10 mg dose	117	27	3.55 (1.07–11.8)	0.038
20 or 40 mg dose	14	2	1.45 (0.24–8.78)	0.681
c.521T/T	119	22		
c.521T/C or C/C	65	10	0.67 (0.32–1.43)	0.301
*ABCG2*				
5 mg dose	53	3		
10 mg dose	117	27	3.56 (1.07–11.8)	0.038
20 or 40 mg dose	14	2	1.53 (0.25–9.34)	0.644
c.421C/C	153	27		
c.421C/A or A/A	31	5	0.81 (0.31–2.10)	0.660
Risk alleles				
5 mg dose	53	3		
10 mg dose	117	27	3.61 (1.09–12.0)	0.036
20 or 40 mg dose	14	2	1.43 (0.23–8.68)	0.700
0–1 alleles	169	31		
2–4 alleles	15	1	0.23 (0.03–1.69)	0.149

CI, confidence interval.

### Fluvastatin and pravastatin

Fluvastatin had 70 users of whom 31 were categorized as statin switchers. No statistically significant association was found for fluvastatin (Table 5). Pravastatin had a total of 92 users of which 29 were categorized as statin switchers. *SLCO1B1* c.521T/C or C/C genotype was associated with an increased risk of pravastatin switching when compared to homozygous reference T/T genotype (HR 2.11, 95% CI 1.01–4.39, *P* = 0.047; Table 6).

**Table 5:** Associations of *SLCO1B1* c.521T>C and *ABCG2* c.421C>A single nucleotide variations, as well as *CYP2C9* phenotypes with intolerance to fluvastatin

Variable	n	Statin switch		
		Events (n)	Hazard ratio (95% CI)	*P*-value
*SLCO1B1*				
20 mg dose	19	7		
40 mg dose	14	6	0.91 (0.31–2.73)	0.868
80 mg dose	37	18	1.62 (0.65–4.05)	0.303
c.521T/T	39	15		
c.521T/C or C/C	31	16	1.42 (0.67–3.01)	0.36
*ABCG2*				
20 mg dose	19	7		
40 mg dose	14	6	0.88 (0.30–2.64)	0.825
80 mg dose	37	18	1.39 (0.58–3.34)	0.466
c.421C/C	61	25		
c.421C/A or A/A	9	6	2.06 (0.84–5.05)	0.113
*CYP2C9*				
20 mg dose	19	7		
40 mg dose	14	6	0.89 (0.30–2.65)	0.832
80 mg dose	37	18	1.39 (0.58–3.34)	0.465
NM	45	14		
PM or IM	25	17	1.36 (0.67–2.78)	0.394
Risk alleles				
20 mg dose	19	7		
40 mg dose	14	6	0.95 (0.32–2.85)	0.928
80 mg dose	37	18	1.65 (0.67–4.04)	0.273
0–1 alleles	52	19		
2–4 alleles	18	12	1.88 (0.90–3.96)	0.094

CI, confidence interval.

**Table 6:** Association of *SLCO1B1* c.521T>C single nucleotide variation with intolerance to pravastatin

Variable	n	Statin switch		
		Events (n)	Hazard ratio (95% CI)	*P*-value
*SLCO1B1*				
20 mg dose	46	9		
40 mg dose	46	20	1.43 (0.65–3.17)	0.376
c.521T/T	59	15		
c.521T/C or C/C	33	14	2.11 (1.01–4.39)	0.047

CI, confidence interval.

## Discussion

In a large observational cohort of statin initiators, we replicated the well-characterized association of *SLCO1B1* c.521T>C with simvastatin intolerance. The effect was consistent in both of our intolerance phenotypes, the first based on statin switching only and the second based on statin switching and temporally associated CK measurement. By using these statin intolerance phenotypes, we also found a significant association of *SLCO1B1* c.521T>C with pravastatin intolerance.

Majority of real-world evidence linking genetic factors with statin intolerance is based on either simvastatin users or at least a majority of simvastatin users due to historically widespread use of simvastatin in favor of other statins. Genetic association studies have rarely stratified for statin type in the primary analyses, in many cases due to insufficient sample size for statins other than simvastatin and in some cases atorvastatin [11,16]. As the pharmacokinetics of statins differ from each other, we found it important, however, to stratify in the primary analyses by statin type in statin-naïve statin initiators. Otherwise, possible associations could be diluted by differences in metabolic and disposition pathways of different statins. For example, common *SLCO1B1* and *ABCG2* variants differentially affect pharmacokinetics of specific statins [6,21–28]. Moreover, simvastatin and atorvastatin are metabolized by CYP3A4/5, and fluvastatin is metabolized by the genetically polymorphic CYP2C9 [9,25,28–31]. A tradeoff of this choice is that we could not account for multiple switches for individual subjects.

Due to the epidemiological nature of our study, the definitions for statin intolerance were based on prescribing patterns (statin switching) or combination of prescribing patterns with clinical chemistry measurements (CK measurements) indicative of intolerance to statin therapy. Our definitions differed somewhat from similar previous studies based on electronic medical records without information about patient-reported symptoms for intolerance [32,33]. We included only statin switching in our phenotype, since in our preliminary analyses with simvastatin-*SLCO1B1* statin switching rather than statin discontinuation or statin dose reduction, best-represented statin intolerance. This choice can be rationalized by the fact that statin discontinuation and dose reduction can occur for multitude of reasons not related to intolerance, which are not possible to be identified from the administrative registry data without access to electronic health records. Thus, statin switching is more likely to represent general statin intolerance but it can also be related to lack of efficacy. Consequently, we found it important to include an intolerance phenotype where change in statin prescribing was temporally associated with another indicator for intolerance, namely a CK measurement. Even though CK measurement can be ordered in various clinical situations, it is typically measured when statin-induced myopathy is suspected. Furthermore, muscle symptoms without CK elevation are a much more common form of statin-related myotoxicity than muscle symptoms with CK elevation [1]. Therefore, we included any CK measurement regardless of result to include also milder cases of myalgia not associated with CK increase. However, it should be noted that in the double-blinded STOMP trial, 9.4% of subjects treated with high dose atorvastatin experienced myalgia, whereas 4.6% of those treated with placebo experienced myalgia even after rechallenge [34]. Thus, our latter phenotype is more likely to represent muscle symptom-based statin intolerance, even though it might not be possible to separate between true statin-induced myalgia and symptoms unrelated to statins. The interval of 365 days between statin purchases for censoring was chosen to be able to catch statin switches after a pause of the initial statin. Our statin intolerance phenotype definitions were corroborated by robust associations of *SLCO1B1* c.521T>C with both intolerance phenotypes in simvastatin users.

In addition to simvastatin, we observed that heterozygous or homozygous *SLCO1B1* c.521T>C carriers had a higher risk of pravastatin intolerance based on statin switching phenotype when compared to reference allele homozygotes. It is noteworthy that the effect was observed in our cohort with low-moderate pravastatin doses as 40 mg is the highest approved dose of pravastatin in Europe rather than 80 mg available in the USA. In a previous pharmacogenetic study of statin efficacy and safety, 143 patients received 10 mg/day pravastatin for 8 weeks followed by 40 mg/day for 8 weeks [16]. Of these patients, 31 experienced a composite adverse event of either premature discontinuation due to any side effect, myalgia, or muscle cramps, or CK elevation during the 16-week follow-up. The *SLCO1B1* c.521T>C SNV showed no association with the composite adverse event. The median follow-up time in our study was more than 2 years in pravastatin initiators, which may partly explain the differing results. Although most statin-induced muscle symptoms occur during the first months of statin usage, myotoxicity can occur even years after statin initiation [7].

We observed no statistically significant increase in statin intolerance risk for atorvastatin, fluvastatin or rosuvastatin with the studied genotypes. For atorvastatin, no signals were evident for genotype-driven increased risk in our cohort of 780 atorvastatin users who most often had 10 and 20 mg prescribed as a starting dose. An earlier meta-analysis of 13 studies with a total of 1550 patients found that the *SLCO1B1* c.521T>C SNV associates with atorvastatin-induced adverse effects [15]. Moreover, in two recent prospective observational studies with substantial sample sizes (870 and 1627), the SNV was associated with an increased risk of atorvastatin intolerance or atorvastatin-associated muscle symptoms [17,32]. In the former study, 90% of the patients were using 80 mg/day atorvastatin, which is a substantially higher proportion than in our study and may partly explain the differing results. Nevertheless, the non-significant HR of 1.25 to 1.3 for the statin switch + CK measurement phenotype in C-allele carriers in our study is close to the odds ratio of 1.44 for atorvastatin intolerance and HR of 1.4 for atorvastatin-associated muscle symptoms in the previous studies [11–13,17,35].

The rosuvastatin cohort consisted of only 184 patients who had most often 5 or 10 mg prescribed starting dose with no tendencies for increased risk in carriers of the candidate risk alleles. The findings are in line with the JUPITER trial in which the *SLCO1B1* genotype was not associated with clinically reported myalgia in a clinical trial setting with rosuvastatin dose of 20 mg [36]. The median follow-up time for rosuvastatin was relatively short, due to a recent increase in its use. This may diminish the sensitivity to identify genotype-intolerance associations.

For fluvastatin, there was a trend towards increased risk of statin intolerance with a point estimate of HR at 1.88 in carriers of 2–4 risk alleles, but perhaps due to low sample size, the finding was not statistically significant. A previous study in renal transplant recipients with 52 cases of fluvastatin-induced adverse muscular or liver symptoms and 52 matched controls, showed that the CYP2C9*2 and *3 alleles and the *ABCG2* c.421C>A SNV significantly increase the risk of these adverse reactions, with odds ratios between 2.4 and 6.6 [37]. Our data suggest that these alleles as well as the *SLCO1B1* c.521T>C SNV may increase the risk of fluvastatin intolerance also among general hypercholesterolemic patients, but further studies are required to confirm the findings.

Our study has some limitations mainly related to its epidemiological and retrospective nature. Obviously, some prescribing changes occurring in our study are not related to statin intolerance and cases of true intolerance cannot be identified from the data. In any case, the study answers the clinical question of how the studied genotypes are related to statin switching. Furthermore, we eliminated obvious causes of confounding in prescribing patterns due to administrative factors by excluding time frames where atorvastatin and rosuvastatin were switched to other statins due to changes in reimbursement policies. The study population consisted of biobank-consented patients with available DNA samples who initiated statin therapy between 1998 and 2016. As the Helsinki Biobank became operational in 2015, the majority of the study subjects had initiated their statin therapy before giving biobank consent and samples to the biobank, presenting a possibility for immortal time bias. For the current study design, this is not of major concern, however, as the study outcome was not related to survival. Moreover, the genotype frequencies observed were in the Hardy-Weinberg equilibrium and match those reported for the Finnish population (Table 1). Lastly, due to low sample size in the fluvastatin, pravastatin, and rosuvastatin cohorts, care is warranted in making conclusions based on the current study alone.

The current study has several clinical implications. First, it corroborates the well-characterized association of *SLCO1B1* genotype with simvastatin intolerance in a real-world setting. Second, despite limited numbers, the *SLCO1B1* genotype associated with pravastatin intolerance at a dose range of 20–40 mg, supporting the role of OATP1B1 activity in pravastatin adverse effects. Third, even though no statistically significant genotype associations were observed for atorvastatin, fluvastatin, or rosuvastatin, these results can be used in future meta-analyses and in compilation of clinically actionable pharmacogenetic guidelines.

In conclusion, *SLCO1B1* c.521T>C genotype was associated with increased switching of simvastatin and pravastatin to other statins in an epidemiological biobank study. The intolerance phenotype derived from statin switching alone or combined with CK measurement appears to be suitable to evaluate statin intolerance. Further studies in larger populations are required to examine the pharmacogenetics of statin intolerance.

## Acknowledgements

We thank all study participants for their participation in the biobank research and the Helsinki Biobank for establishing the study cohort.

This study was supported by grants from the European Research Council (Grant agreement 725249), State funding for university-level health research, and the Sigrid Jusélius Foundation (Helsinki, Finland).

### Conflicts of interest

There are no conflicts of interest.
